# A Lesion on the “King of Kings”: Neurofibromas in the Parthian Empire’s Arsacid Dynasty

**DOI:** 10.7759/cureus.46248

**Published:** 2023-09-30

**Authors:** Matthew D Turner, Adam Sunday

**Affiliations:** 1 Emergency Medicine, Penn State Health Milton S. Hershey Medical Center, Hershey, USA; 2 Emergency, Penn State Health Milton S. Hershey Medical Center, Hershey, USA

**Keywords:** dermatology, trichoepithelioma, history of medicine, ancient history, parthia, neurofibromatosis 1

## Abstract

Multiple kings of the Arsacid Dynasty of the ancient Parthian Empire are depicted on their coinage with a recurrent facial lesion, one that is found across multiple generations. Multiple theories have attempted to explain this phenomenon, from basal cell carcinoma to hereditary trichoepithelioma. In this paper, we suggest that these lesions are possibly a representation of the neurofibromas found in Neurofibromatosis 1, an autosomal dominant disease process.

## Introduction and background

Introduction

For 500 years, the great Parthian Empire stretched from Syria to India in much of what is now modern Iran, at the intersection of East and West, simultaneously trading with both China and the Roman Empire via the Silk Road. The empire was founded in approximately 250 BC by the legendary Arsaces I, from whom the empire’s ruling dynasty - the Arsacids - would take their name. Unfortunately, while the Parthian Empire was a mighty force and successfully defeated the neighboring Roman Empire on several memorable occasions, little is ultimately known about the Arsacid Dynasty or the empire that they ruled for half a millennium. Archaeological evidence is scant, and written records even more so [1]. Most documentation about the Parthian Empire comes from either Greek, Roman, or Chinese literary sources - many of which suffered from inherent biases and distance, both geographic and chronological, from the events they described [1].

Since almost “all known records on the history of Parthia originated outside the country” [2], one of the most valuable sources of information about the Arsacid Dynasty and the Parthian Empire comes from the coins that they minted [1]. The right to mint coinage was not solely that of the reigning monarch; many regions of the Empire and even individual cities were granted the right to mint their coins, which provided priceless “hints about the internal history of the Arsacid state” [2]. The silver drachm was traditionally the main currency, based on a standard of approximately 4 grams of silver for most of the empire’s history [3].

## Review

Nodules

**Figure 1:**
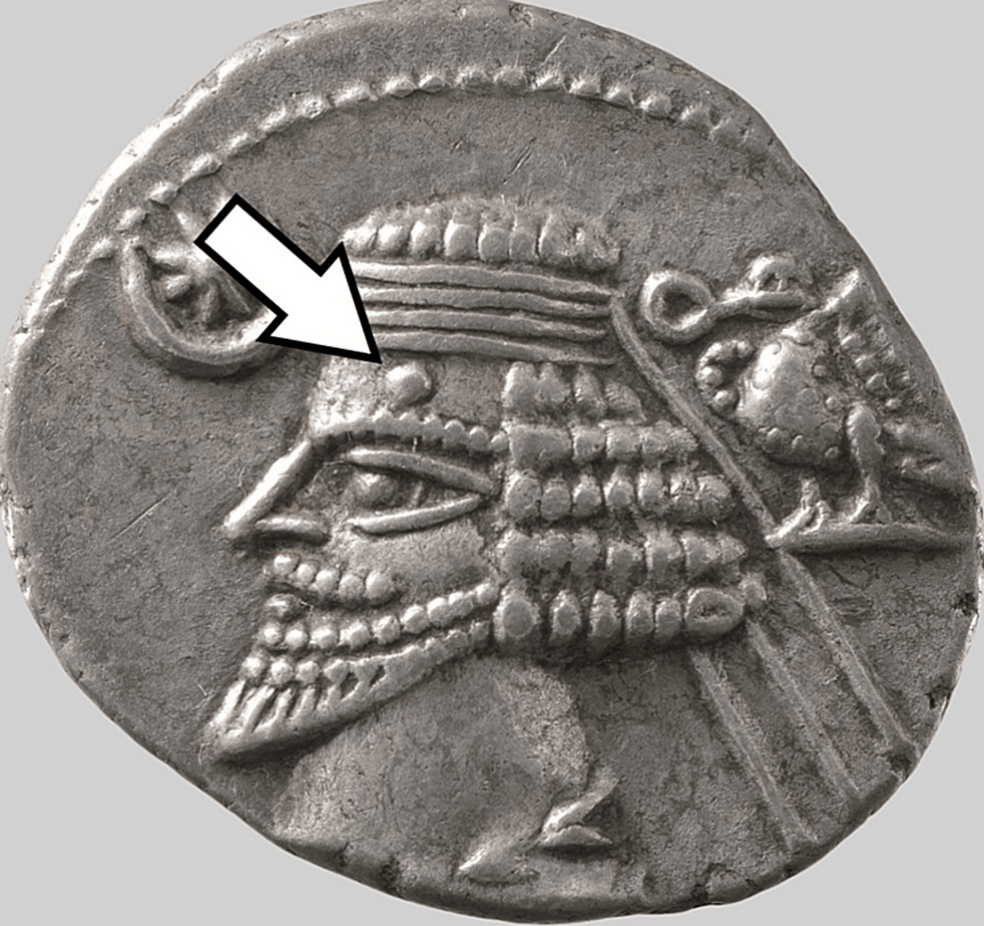
A silver drachm depicting Phraates IV. Note the nodule on the king’s forehead. This picture was obtained from the Metropolitan Museum of Art and is part of the public domain.

Hart’s 1966 paper on the subject was one of the first to note the unusual nodule - a “wart” that appears on the official coinage of Mithradates II [4]. One of the greatest kings of the Parthian Empire, Mithradates II ruled from 122 to 91 BC and oversaw a massive expansion of the empire’s power, going so far as to adopt the title “King of Kings” in 109 BC [2]. He initiated diplomatic relations with Rome, establishing an “increasingly frequent and more formal” relationship between the two powers that is responsible for much of our current knowledge of the empire today [2]. Mithradates II was succeeded by Sinatruces, who rose to the throne after a period of unrest in 88/87 BC. Phraates III (71/70-58/57 BC) succeeded Sinatruces. Phraates III was unseated in 58/57 in a coup by his sons, Orodes II and Mithradates III, who subsequently turned upon one another, with Orodes ultimately emerging as “the final victor” [2]. While none of the intervening monarchs had shown any evidence of nodules on their coinage, Orodes II is depicted with a nodule on his forehead, similar to that of his ancestor, Mithradates II [4]. Orodes II relinquished power to his son, Phraates IV, in 38 BC [2]. Of note, one of Orodes’ other sons, Pacorus I, has also been depicted on official coinage with a faint lesion on his cheek. Phraates IV had “many coins … which show a prominent lesion on the forehead” [4], as shown in Figure 1 [5].

Phraates V, also known as Phraataces (3/2 BCE-2 CE) succeeded his father [2]. Although his reign was not a long one, the coins that we have of him “suggest the presence of a periauricular nodule but are not definite” [4].

**Figure 2:**
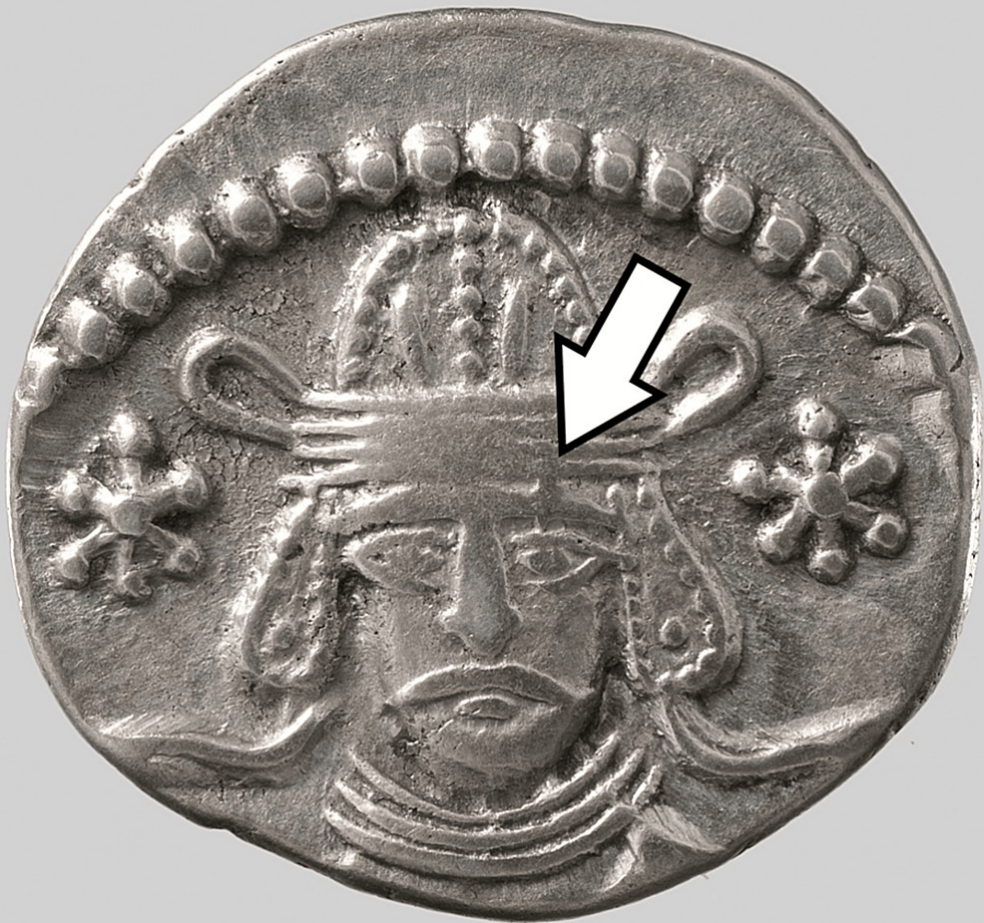
A silver drachm of Vardanes. Note the nodule on the king’s forehead. This picture was obtained from the Metropolitan Museum of Art and is part of the Public Domain.

Several years of conflict followed, until Artabanus II, “a member of the sideline of the Arsacids” rose to the Parthian throne. Unlike Phraates V, he was not descended from Phraates IV [2] and claimed descent from the Arsacid dynasty through his mother’s line. One of the coins depicting him “shows a lesion on the midforehead” [4]. Artabanus’ son Vardanes I (39-45 CE) followed his father [2] and was depicted with a “lesion on the left forehead” [4], as seen in Figure 2 [6]. His brother, Gotarzes, another claimant to the throne, is depicted with a “conspicuous lock of hair over the left forehead region”, a depiction that Hart suggests was used to cover a similar facial lesion [4].

Another period of political strife followed Vardanes’ reign until his first cousin Vologases I (51-78/79 CE) ascended the throne [2]. Like many of the earlier kings of Parthia, he is depicted with a prominent lesion on his forehead. However, his son Pacorus II (78-110 CE) [2] is depicted with no lesions on his official coinage [4]. Vologases II (110-147 CE), son of Pacorus and grandson of Vologases I, is depicted with a “nodule on the forehead” with some of his coinage even displaying the “cosmetic lock of hair” seen in Gortazes [4].

**Figure 3:**
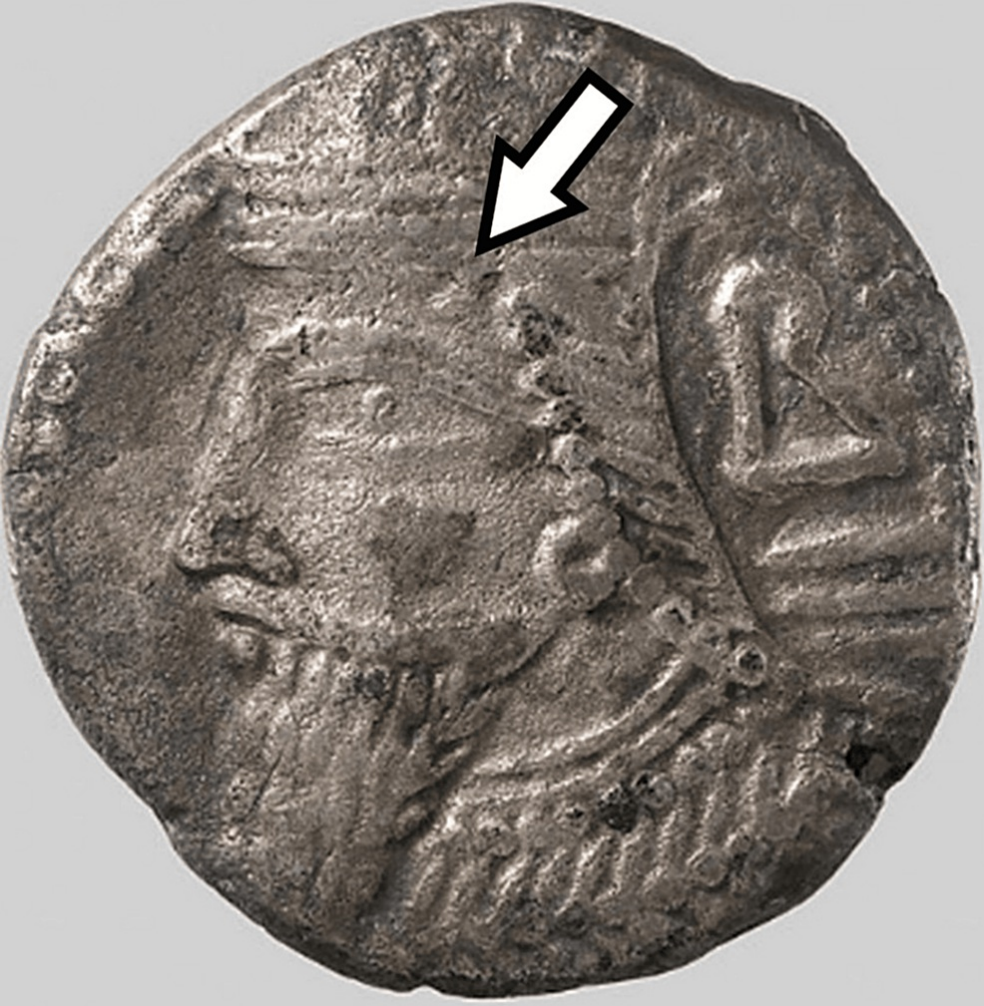
A tetradrachm coin of Vologases V. Note the forehead nodule. This picture was obtained from the Metropolitan Museum of Art and is Public Domain.

Vologases III (147-191/192), son of Vologases II, has a similar “temporal lock of hair which may indicate an underlying lesion” [4]. His son Vologases IV (191-207/208) is depicted with “a nodule on each side of the forehead”, and his successor Vologases V (207/208-221/222) “has the temporal lock” [4], as seen in Figure 3 [7]. The empire shortly disintegrated after the reign of Vologases V; there is little coinage of the final kings of the Arsacid Dynasty available to us today [4].

While the “Parthian state was an absolute inherited monarchy with a traditional principle of passing down the rule to the eldest son” [2], the lineage of the Arsacid Dynasty can be difficult to track; the Arsacids often practiced polygamy, and in the cases of Artanabus II and Vologases I, it was a member of a minor branch of the family that rose to the throne [2]. Further complicating the matter is that the Parthian royal family, much like other contemporaries in the region including the Ptolemies and Seleucids, often practiced sibling marriage [8]. Mithradates II, the founder of the dynasty, married two of his half-sisters, setting the stage for “marriage among relatives and even siblings [to be] possible and permitted at the royal court” [1]. His eldest son, Gotarzes I, one of the claimants to the throne in a bloody civil war, is also recorded as being married to two of his sisters simultaneously. Orodes I, a later Parthian king, was noted by foreign sources to rule Parthia with his sister-wife Ispubarza [9]. In one notable example, Queen Mousa, the wife of Phraates IV, allegedly helped her son Phraates V overthrow her husband before marrying her son [10,11]. The commonality of this inbreeding, along with the scant written records available to us from the Parthian Empire [1], makes the precise lineage of the Arsacid Dynasty quite difficult to track.

However, the commonality and recurring image of the “wart” seen on the coinage of the Parthian kings has led several researchers to propose that these lesions are hereditary in nature [12]. It is unlikely that these lesions were meant to be symbolic - the imagery of the kings is meant to be “characteristic and realistic,” with identifiable changes in hairstyles and facial hair concurrent with the times [13]. In addition to this, the stylistic “furrowing of the brows, parting of the lips, and placing a groove on either side of the mouth” and occasional protruding ears displayed on the coins suggest a high likelihood of realism in the depiction of the kings [12]. These nodules clearly “represented … an important (distinctive) facial feature” that may have also helped strengthen the legitimacy of the dynasty’s reign [14].

Basal cell carcinoma

 It has been suggested that the initial lesion on the coinage of the Parthian kings - that of Mithradates II (123-88 BC) may be due to a basal cell carcinoma on the lateral aspect of the king’s left lower eyelid [15]. While this is certainly possible in an isolated case, the sheer frequency of similar lesions in the king’s descendants, stretching for multiple generations, suggests to us that Mithradates’ lesion was more likely due to a hereditary etiology.

Trichoepithelioma

In his 1966 paper, Hart raises the possibility that the recurrent lesions seen on the kings of Parthia may be due to trichoepithelioma, “a benign tumour [sic] which occurs chiefly on the forehead, around the nose and in front of the ear” [4]. These benign tumors of the hair follicles were first identified in 1892 and may manifest in a hereditary or non-hereditary form [16].

In the hereditary form, multiple familial trichoepithelioma (MFT) is associated with either a mutation on the tumor suppressor encoding gene chromosome 9q21 [16] or the cylindromatosis tumor suppressor gene (CYLD) on chromosome 16q12-13 that is transferred in an autosomal dominant fashion, and typically arises in “young to aging adults” [16]. MFT will often manifest as “multiple skin colored, pink, or bluish firm, rounded, translucent, shiny, well-demarcated papules or nodules” [16]. Typically, these lesions are 2-8 mm in diameter [17]. As Hart also points out, these lesions are predominantly found on the face, particularly around the nose, forehead, and eyelids [4,16]. These lesions are typically asymptomatic, and largely treated only for cosmetic concerns [16]. In the literature, families have been described with MFT presenting in at least four consecutive generations [17,18]. Often, these lesions will not present until puberty or later in life [17].

Unfortunately, Hart’s hypothesis suffers from a significant flaw. MFT “seems to affect females more, probably due to smaller expressivity and chromosomal penetration in males” [16]. Multiple studies have found that while “both genders receive the gene equally, most patients are young to aging adult women due to lessened expressivity and penetrance in men” [12,17]. As discussed earlier, the Arsacid Dynasty did have a high degree of consanguinity and often practiced sibling marriage, similar to other neighboring powers in the region at that time [8]. The reduced genetic diversity that results from inbreeding may lead to the homozygosity of previously low-penetrance cancer genes [19]. In this context, the high degree of consanguinity of the Arsacid Dynasty may have resulted in an unusually high penetrance of MFT in the male lineage.

However, while this explanation is a distant possibility, not all the marriages of Parthian kings were consanguineous - many kings married both domestic and foreign nobility in order to strengthen their political standing at home and abroad [1]. The empire also did not always pass directly down the patrilineal line; on at least two occasions, distant branches of the family rose to the throne [2]. Given this, it is doubtful that the Arsacid Dynasty ever reached a level of inbreeding necessary to significantly alter the baseline penetrance of MTF - at least to the degree that would require it to present across multiple generations for centuries.

Neurofibromatosis 1: the most likely etiology

Todman’s 2008 proposal that the hereditary lesions of the Arsacid Dynasty were due to neurofibromatosis remains the strongest theory. Neurofibromatosis includes neurofibromatosis type 1 (NF1) and neurofibromatosis type 2 (NF2), which are both autosomal dominant and share common clinical features “including hyperpigmented birthmarks of the skin” [13]. It is possible that the solitary round nodule seen on the Parthian kings’ imagery may be suggestive of a hereditary neurofibroma such as in NF1 [13]. NF2 is less likely as a possibility, due to both its rarity and increased severity as a disease - often manifesting with substantial hearing loss secondary to bilateral vestibular schwannomas [20].

NF1, also known as von Recklinghausen disease, is an autosomal dominant pathology due to a mutation of the NF1 gene on chromosome 17q11. Unlike MFT, it has nearly “100 percent penetrance by adulthood”, with no significant difference in gender [21]. Nearly all patients with NF1 display “benign cutaneous and subcutaneous neurofibromas” with a number that may range from “only a few to hundreds or more” [21]. 95% of patients with NF1 will develop these “discrete benign neurofibromas within the dermis” [20]). These neurofibromas typically develop between 10 and 20 years of age [21]. However, while penetrance is up to 100%, the expression of neurofibromas may have significant clinical variations, even within close relations [20]. Generally, the cutaneous neurofibromas associated with NFI “do not become apparent until puberty and may continue to increase in size and number throughout adulthood” [22], consistent with Hart’s observation that the coins of Arsaces I from early in his reign do not display the lesion seen in his later reign, suggesting “that the tumour [sic] appeared after he ascended the throne” [4].

Diagnosis of NF1 usually relies on the patient having two or more of the following symptoms: six or more café-au-lait spots, 1.5 cm or larger neurofibromas post-puberty, axillary or groin freckling, or two or more neurofibromas of any type [20]. Unfortunately, due to the limited evidence available to us from the primary sources, it is impossible to make a diagnosis from the imagery found on the Parthian coins. One of the weaknesses of this theory is that most of the coins that have been discovered only feature a solitary nodule, which is not consistent with NF1. However, this may simply be due to a lack of space for a proper artistic depiction - it is possible that “the depiction of just one nodule on a small coin portrait may be representative of multiple such lesions” [12].

Further strengthening the NF1 theory is evidence of other artistic depictions of neurofibromatosis from the Hellenistic era - researchers have identified statues with “multiple skin nodules on the torso and limbs consistent with Neurofibromatosis” [12]. The higher degree of penetrance displayed by NF1 also makes it a more likely candidate than trichoepithelioma, even when accounting for the high rates of consanguinity present in the Arsacid Dynasty.

## Conclusions

The recurrent lesion found on multiple generations of the Arsacid Dynasty is possibly a form of neurofibroma secondary to the autosomal dominant, high-penetrance disease NF1. It is unlikely that the lesion depicted on the Parthian Empire's coinage is meant to be figurative or symbolic, given the otherwise realistic depiction of the kings. Given the high degree of expressivity of the lesion across multiple generations of men, the lower-penetrance form of hereditary trichoepithelioma is significantly less likely, even when accounting for the consanguinity often found at the Parthian court. While it is impossible to fully determine, it is possible that NF1 is responsible for the lesions seen across generations of this ancient dynasty.
